# Expectations and educational needs of rheumatologists, rheumatology fellows and patients in the field of precision medicine in Canada, a quantitative cross-sectional and descriptive study

**DOI:** 10.1186/s41927-021-00222-2

**Published:** 2021-11-29

**Authors:** Sophie Ruel-Gagné, David Simonyan, Jean Légaré, Louis Bessette, Paul R. Fortin, Diane Lacaille, Maman Joyce Dogba, Laëtitia Michou

**Affiliations:** 1 Division of Rheumatology-R4774, Department of Medicine, CHU de Québec-Université Laval, 2705 Boulevard Laurier, Quebec, QC G1V 4G2 Canada; 2 Centre de Recherche du CHU de Québec-Université Laval, Quebec, Canada; 3 Arthritis Research Canada, Quebec, Canada; 4 Arthritis Research Canada, University of British Columbia, Vancouver, BC Canada; 5 Department of Family and Emergency Medicine, Faculty of Medicine, Université Laval, Quebec, Canada; 6 Centre de Recherche en Santé Durable VITAM, Quebec, Canada

**Keywords:** Precision medicine, Personalized medicine, Pharmacogenomics, Biomarker, Rheumatology, Education, Patient decision-making

## Abstract

**Background:**

Precision medicine, as a personalized medicine approach based on biomarkers, is a booming field. In general, physicians and patients have a positive attitude toward precision medicine, but their knowledge and experience are limited. In this study, we aimed at assessing the expectations and educational needs for precision medicine among rheumatologists, rheumatology fellows and patients with rheumatic diseases in Canada.

**Methods:**

We conducted two anonymous online surveys between June and August 2018, one with rheumatologists and fellows and one with patients assessing precision medicine expectations and educational needs. Descriptive statistics were performed.

**Results:**

45 rheumatologists, 6 fellows and 277 patients answered the survey. 78% of rheumatologists and fellows and 97.1% of patients would like to receive training on precision medicine. Most rheumatologists and fellows agreed that precision medicine tests are relevant to medical practice (73.5%) with benefits such as helping to determine prognosis (58.9%), diagnosis (79.4%) and avoid treatment toxicity (61.8%). They are less convinced of their usefulness in helping to choose the most effective treatment and to improve patient adherence (23.5%). Most patients were eager to take precision medicine tests that could predict disease prognosis (92.4%), treatment response (98.1%) or drug toxicity (93.4%), but they feared potential negative impacts like loss of insurability (62.2%) and high cost of the test (57.5%).

**Conclusions:**

Our study showed that rheumatologists and patients in Canada are overall interested in getting additional precision medicine education. Indeed, while convinced of the potential benefits of precision medicine tests, most physicians don’t feel confident in their abilities and consider their training insufficient to incorporate them into clinical practice.

**Supplementary Information:**

The online version contains supplementary material available at 10.1186/s41927-021-00222-2.

## Background

Precision medicine, as a personalized medicine approach based on biomarkers, is a booming field. In recent years, there have been several global initiatives such as the Human Genome Project, the 100,000 Genomes Project and the US Precision Medicine Initiative to study the role of genetic, environmental and behavioural factors in disease to offer personalized care [1]. Pharmacogenomics, a discipline that studies the role of genes in drug response and drug toxicity, is an important part of precision medicine [2]. Some tests are already widely prescribed by rheumatologists, for instance the identification of HLA-B27 allele in spondyloarthropathies [3] or the anti-CCP in rheumatoid arthritis, the latter having prognosis and treatment response prediction utility in addition to its diagnosis utility. The measurement of thiopurine methyltransferase (TPMT) activity before the use of azathioprine is frequently used to identify patients at high risk of drug toxicity [4]. The genetic search of the HLA-B5801 has also demonstrated a clinical utility to identify patients with increased risk of severe adverse reactions to allopurinol. Many clinical biomarkers are in development and expected to better guide treatment choices, by considering inter-individual variability and the influence of genetics polymorphisms in response to some conventional synthetic disease-modifying anti-rheumatic drugs (csDMARDs) or biological therapies [5–7].

We did not find any study evaluating the knowledge, experience, expectations and educational needs of rheumatologists and rheumatology fellows in the field of precision medicine in the literature. In contrast, several studies have already been conducted with primary care physicians. These studies reveal that generally, primary care physicians have a positive attitude toward precision medicine but that their knowledge and experience are modest [8, 9]. It is therefore essential to better equip rheumatologists and rheumatology fellows so that they are more comfortable in prescribing, interpreting and explaining precision medicine tests to their patients. Other studies have focused on the knowledge and expectations of patients, but again, none has been done in rheumatology. They demonstrated that patients generally have a more favourable attitude than doctors toward the integration of precision medicine into clinical practice, but that their knowledge and understanding of genetic principles and tests are limited [8, 9]. It is therefore essential to better inform patients so that they can consent in a free and informed manner to having these tests. Furthermore, it is imperative to explain the significance of a test result and its implication so that the patient may understand how to integrate these into their decision-making. This project assessed the expectations and educational needs in the field of precision medicine among rheumatologists, rheumatology fellows and patients with rheumatic diseases in Canada.

## Methods

We conducted a quantitative cross-sectional and descriptive study consisting of an anonymous online survey lasting about 15 min for rheumatologists and fellows and another anonymous online survey lasting 10 min for patients. Both surveys were available in French and in English (English versions are enclosed in the Additional files 1 and 2). The CHU de Québec—Université Laval Ethics Committee approved this study (project 2018-4065) and all participants gave an implied consent by completing the anonymous survey.

### Surveys

*For rheumatologists and fellows*, after a few socio-demographic questions, the survey focused on the following themes, using mostly single or multiple-choice questions or Likert scales: precision medicine knowledge and experience (22 questions), expectations and educational needs (8 questions) and the preferred format for training (2 questions). Optimal utilization of biomarkers was evaluated through four real-life clinical vignettes (HLA-B27, Anti-cyclic citrullinated peptide (anti-CCP), TPMT and HLA-B*5801) and one fictive clinical vignette consisting of a companion test before a novel biological agent prescription. Since we did not find any standardized questionnaire on the use of precision medicine applying to rheumatology in the medical literature, we developed our surveys.

*For patients*, after collecting socio-demographic data, the same themes as for physicians (6 questions on experience, 13 questions on expectations, 4 questions on educational needs) were explored with a focus on concerns about test results, impact on treatment and on lifestyle, follow-up, and access to data, using mostly single or multiple-choice questions or Likert scales.

Cover letters and surveys were developed in French and then translated into English. The understanding of both surveys was evaluated in French and in English by the research team members, which gave rise to several changes in the English versions of both surveys to facilitate the understanding after translation from the French version. The understanding of the survey in French for patients was also evaluated by four patient partners, members of the Patients Interested in Research on Arthritis (PIRA) group from our institution and affiliated with Arthritis Research Canada. The test–retest reliability of the final French version of both surveys was assessed in a sample of four medicine residents and four patients from the PIRA group. The reliability testing consisted of a duplicate administration of the whole survey for patients 1 week apart to assess the consistency of the answers. For the rheumatologists and fellow’s survey, the reliability testing consisted of a duplicate administration 1 week apart performed by medicine fellows only for the precision medicine knowledge and precision medicine expectations sections of the survey. The reliability testing resulted in minor modifications to the patient survey.

### Population

*Rheumatologists and fellows* With the authorization of the Canadian Rheumatology Association (CRA), the 519 adult and pediatric rheumatologists and about 60 rheumatology fellows, all members of the CRA were invited to participate to the anonymous survey. They received an email from the CRA containing a cover letter and the Internet link to access the consent form followed by the electronic anonymous survey. This email was followed by two reminders at intervals of 1 month.

*Patients* The source population consisted of patients 18 years or older, suffering from a rheumatic disease followed up by a rheumatologist across Canada. Patients were recruited by an email sent by The Arthritis Society, Arthritis Research Canada and the Arthritis Consumer Expert to their members, containing a cover letter and the Internet link to access the consent form followed by the electronic anonymous survey. This email was followed by two reminders at intervals of 1 month.

### Sample size

We calculated that 222 rheumatologists, 52 fellows and 385 patients completing the surveys were needed to estimate the proportion of participants with one specific need in each group assuming a margin of error of 5% with a confidence level of 95%, and a finite population of 519 and 60 respectively for the first two groups.

### Statistical analysis

Both survey data were collected in an excel database by the LimeSurvey tool itself. Descriptive analyses were performed using frequencies, percentages, and 95% confidence interval for binomial proportions. For the test–retest reliability of the surveys, the consistency of the survey’s answers was assessed using simple or weighted Cohen’s kappa coefficients. The reliability of the questions on a Likert-type scale (ranging from 1 to 5) was assessed by Cronbach’s alpha. The Pearson’s chi-squared tests (or Pearson’s exact tests, when appropriate) were used to estimate associations between socio-demographic data and survey answers. Pairwise deletion (available-case analysis) was used to handle missing data which was considered as missing at random. Socio-demographic data of rheumatologists and patients were compared to recently published data to assess whether our survey respondents were representative of the Canadian population of rheumatologists and RA patients using chi-square tests for single proportion [10, 11]. Statistical analyses were performed using SAS Statistical Software v.9.4 (SAS Institute, Cary, NC, USA), with a two-sided significance level set at *p* < 0.05. We performed a qualitative description of free comments provided by study participants at the end of both anonymous surveys regarding suggestions on how training in the field of precision medicine could be improved. All comments were compiled and similar ideas were then regrouped.

## Results

### Description of the rheumatologists and fellows who responded to the survey

45 rheumatologists and 6 fellows participated in this study, and 34 of them completed the whole survey. 51% of respondents were male, 40% were 35–49 years old, 36% were from British Columbia, 35.6% had been practising rheumatology for more than 20 years, mostly in University hospital (64%) and patient care was their principal activity (86%) (Additional file 3: Table S1).

### Samples representativeness for rheumatologists and fellows

Our sample of rheumatologist was representative of the Canadian rheumatologists for sex, age and number of years of medical practice. The province where rheumatologists practised in our sample was representative of the provincial distribution of rheumatologists for Alberta, Quebec and the Atlantic provinces, but there were more participants from British Columbia and fewer from Ontario than expected (Additional file 3: Table S2).

### Reliability of results for rheumatologists and fellows survey

Among rheumatologists and fellows, the consistency of the survey’s answers was excellent (κ = 1) for 7 questions, moderate (κ ranging from 0.4 to 0.57) for 9 questions, fair (κ = 0.25) for one question, slight to poor (κ ≤ 0) for 8 questions (Additional file 3: Table S3). Cronbach's alpha for 7 questions about knowledge on precision medicine was 95%, for 7 questions on potential benefits of precision medicine was 88%, and for 11 questions evaluating barriers to use precision medicine was 76%.

### Rheumatologist and fellows’ educational needs in the field of precision medicine

25 rheumatologists and rheumatology fellows (78.1%) answered they would like to receive additional training on precision medicine in rheumatology. Their main topics of interest were the clinical utility of precision medicine tests (100% of respondents), their validity and accuracy (100%), the interpretation of test results (100%) and strategies for integrating these tests into health care practice (100%) (Additional file 3: Table S4). Their favourite teaching methods were conferences (96.3%), small group workshops with clinical scenarios (88.5%), self-learning modules (79.2%) and web sites (70.8%) (Additional file 3: Table S5) and 24 participants (92.3%) mentioned they would prefer a combination of methods. For the 7 rheumatologists and fellows (21.9%) who were not interested in pursuing precision medicine education, the main reasons reported being a lack of evidence-based guidelines supporting the use of precision medicine tests in rheumatology, a lack of availability of those tests, the wide dissemination of written information on the subject and the fact that they were already comfortable with the use of those tests. We did not observe any statistically significant association between demographic variables and an interest in pursuing additional training (data not shown). The descriptive analysis of the seven open-ended comments on medicine precision training revealed that this training should start in medicine school and that the development of chart audit tools to perform practice reflection exercise should be useful. Participants also estimated that more research presentations and workshops are needed.

### Rheumatologist and fellows’ experience in the field of precision medicine

38 participants (84.4%) mentioned they had heard about precision medicine tests before and 19 (42.2%) had already prescribed them to their patients. The most prescribed tests were rheumatoid factor, anti-CCP antibodies, antibodies panels for lupus, myositis and scleroderma, HLA-B27, HLA-B51, HLA-B*5801, TPMT and G6PD deficiency. Only 6 rheumatologists and fellows (13.6%) reported having received training in precision medicine as part of their university medical education and 17 (38.6%) having received training in other circumstances, mostly reading of articles in scientific journals (76.5%) and in medical continuing education conferences (70.6%). Most participants (72.1%) judged their training insufficient to integrate precision medicine tests into their current medical practice.

### Rheumatologist and fellows’ knowledge in the field of precision medicine

The analysis of clinical vignette answers showed that knowledge on how to use precision medicine tests in clinical practice is mostly good except for a poorer performance for the question on indications of HLA–B*5801 test (Table 1). Many rheumatologists and fellows, however, could not report which type of tests (genotyping, phenotypic test or enzymatic activity) were available in their workplaces (65% for HLA-B27 and 54.3% for TPMT). Their preferred method for reporting test results, was risk categories (35.5%) followed by Fagan nomogram and dichotomous results (29.1% for both methods). When self-assessing their knowledge, about half of the participants felt confident in their global capacity to use precision medicine tests in the clinical setting (Table 2) but they seemed less comfortable identifying which tests were available in their workplace, prescribing the tests, counselling patients on the risks, benefits and limitations of those tests and communicating the test results to their patients.

**Table 1:** Evaluation of knowledge on precision medicine of rheumatologists and fellows by the use of clinical vignettes#

Vignette topics	Correct answers, N (%)*
Utility of HLA-B27 testing for children of people with ankylosing spondylitis	33/34 (97.1)
Utility of anti-CCP antibodies research for patient with suspicion of rheumatoid arthritis	
Diagnosis utility	27/36 (75)
Prognosis utility	30/36 (83.3)
Treatment response prediction	14/36 (38.9)
Absence of utility for toxicity prediction	36/36 (100)
Utility of TPMT test before starting treatment with azathioprine	
Absence of utility for identification of patients who are likely to respond to azathioprine	36/36 (100)
Absence of utility for identification of patients who are unlikely to respond to azathioprine	34/36 (94.4)
Utility for identification of patients who are likely to develop neutropenia associated with azathioprine use	28/36 (77.8)
Populations on which to perform HLA–B* 5801 genetic test before starting allopurinol	
Thai	8/36 (22.2)
Han Chinese	20/36 (55.6)
Korean with stage G3a chronic kidney disease or worse	8/36 (22.2)
Utility of HLA–B* 5801 genetic test before starting allopurinol	
Absence of utility for identification of patients who will need higher dosage of allopurinol to reach efficacy	36/36 (100)
Absence of utility for identification of patients who are unlikely to respond to allopurinol	36/36 (100)
Utility for identification of patients who are likely to develop a hypersensitivity reaction to allopurinol	23/36 (63.9)

%*Percentage after excluding missing values

^#^Clinical vignettes are presented in the Additional file 2 in the survey for rheumatologists in pages 3–6

**Table 2:** Self-Assessment of knowledge in precision medicine according to rheumatologists and fellows (n = 34)

Topics	Strongly agree or agree N (%)*	Neutral N (%)*	Disagree or strongly disagree N (%)*
Capacity to identify clinical situations in which tests are useful	20 (58.9)	9 (26.5)	5 (14.7)
Awareness of tests availability in my workplace	16 (47.1)	9 (26.5)	9 (26.5)
Comfort with prescribing tests	15 (44.1)	10 (29.4)	9 (26.5)
Confidence in capacity to provide quality counselling about the risks, benefits and limitations of tests	16 (47.1)	12 (35.3)	6 (17.7)
Comfort with interpreting test results	19 (55.9)	12 (35.3)	3 (8.8)
Confidence in capacity to communicate tests results and answer patients questions	19 (55.8)	9 (26.5)	6 (17.6)
Comfort with recommending a treatment based test results	15 (44.2)	15 (44.1)	4 (11.8)

%* Percentage after excluding missing values

### Rheumatologist and fellows’ expectations about precision medicine

Most participants agreed that precision medicine tests are relevant to current medical practice and likely to change clinical approach with benefits such as helping to determine prognosis, diagnosis and prevent treatment toxicity. They were, however, less convinced that precision medicine tests are useful in helping to choose the most effective treatment and improve patient adherence (Table 3). We observed that rheumatologists and fellows who had previously prescribed precision medicine tests for their patients were more likely to have a positive perception of the usefulness of those tests. In addition, those who had received specific training on precision medicine were more likely to have a positive perception of their ability to use it in their clinical practice. The main barriers identified to the implementation of precision medicine tests in medical practice were limited availability, cost, delay in obtaining results and the impact of the results on their patient’s insurability (Table 4).

**Table 3:** Benefits of precision medicine according to rheumatologists and fellows (n = 34)

Potential benefits of precision medicine	Strongly agree or agree N (%)*	Neutral N (%)*	Disagree or strongly disagree N (%)*
Relevant to current medical practice	25 (73.5)	6 (17.6)	3 (8.8)
Susceptible to change clinical approach	24 (70.6)	9 (26.5)	1 (2.9)
Useful to determine prognosis	20 (58.9)	11 (32.4)	3 (8.8)
Useful for diagnosis purpose	27 (79.4)	6 (17.6)	1 (2.9)
Useful to choose the most effective treatment	17 (50.0)	10 (29.4)	7 (20.5)
Useful to avoid treatment toxicity	21 (61.8)	9 (26.5)	4 (11.7)
Useful to improve patients’ compliance	8 (23.5)	15 (44.1)	11 (32.3)

%* Percentage after excluding missing values

**Table 4:** Barriers to the implementation of precision medicine according to rheumatologists and fellows (n = 34)

Potential barriers to implementation of precision medicine	Strongly agree or agree N (%)*	Neutral N (%)*	Disagree or strongly disagree N (%)*
Not enough scientific evidence	12 (35.3)	10 (29.4)	12 (35.3)
Lack of guidelines on precision medicine	15 (44.1)	10 (29.4)	9 (26.5)
Limited availability of tests	21 (61.7)	11 (32.4)	2 (5.9)
Cost of tests	20 (58.8)	11 (32.4)	3 (8.8)
Delay obtaining test results	20 (58.8)	9 (26.5)	5 (14.7)
Insufficient personal knowledge	10 (29.4)	12 (35.3)	12 (35.3)
Confidentiality of test results	5 (14.7)	13 (38.2)	16 (47.1)
Impact of test results on patient’s job	7 (21.2)	12 (36.4)	14 (42.5)
Impact of test results on patient’s insurability	20 (58.9)	8 (23.5)	6 (17.6)
Impact of test results on patient’s anxiety	16 (47.1)	12 (35.3)	6 (17.6)
Impact of test results on patient’s family members	15 (44.2)	12 (35.3)	7 (20.5)

%* Percentage after excluding missing values

### Description of the patients who responded to the survey

All in all, 277 patients participated to this study, 208 of them answered the entire questionnaire. 84% were female, 45.2% were 50–64 years old, 58% were married, 73.9% had children and 54.4% had a university degree. Most respondents were living in Quebec (33.5%) or Ontario (26.8%), 48.2% had a diagnostic of RA, 88.5% were taking medication for their rheumatic disease and 60.7% had comorbidities (Additional file 3: Table S6).

### Sample representativeness for patients

Among the patients, the proportion of women in our sample was greater than in the Canadian population suffering from rheumatic diseases and people 65 years or older were underrepresented. The provincial distribution of our sample was representative for most provinces; however, we had more participants from British Columbia and Quebec and fewer from Ontario (Additional file 3: Table S7).

### Reliability of results for patients’ survey

In patients, the concordance for demographic questions was excellent (κ = 1) for 7 questions, moderate (κ = 0.5) for one question and fair (κ = 0.33) for 2 questions. For the questions on experience, expectations and educational needs, the consistency of the survey’s answers was excellent (κ = 1) for 12 questions, substantial (κ ranging from 0.6 to 0.71) for 4 questions, moderate (κ ranging from 0.42 to 0.6) for 4 questions, fair (κ = 0.33) for one question and slight (κ = 0) for 3 questions (Additional file 3: Table S8). Cronbach's alpha for 3 questions about acceptability of precision medicine tests was 79%, and for 10 questions measuring sources of concern of patients toward precision medicine was 87%.

### Patients educational needs in the field of precision medicine

198 patients (97.1%) mentioned they would like to receive additional information on precision medicine in rheumatology. Their preferred educational modalities were web site (92.3% of respondents), videos/podcasts (85.9%) and self-learning modules (83.4%) (Additional file 3: Table S9). They also suggested emails, educational brochures, social media and discussion with their physicians as other interesting methods to acquire new information. The descriptive analysis of the 61 open-ended comments on medicine precision training provided a critical need for patients to have much more access to information on precision medicine, in an understandable manner and without any bias of manufacturers of the tests. Many patients prefer having information through their rheumatologist or through brochures in their rheumatologist’s waiting room. Several patients have suggested that the education material should be co-developed by physicians together with patients or patient associations.

### Patients experience in the field of precision medicine

Only 30 patients (14.1%) answered that they had ever taken a precision medicine test and 13 (6%) had a family member who had taken a test, mostly HLA-B27 testing and BRCA gene mutation research for breast cancer. Nevertheless, most participants felt confident in their ability to understand the usefulness (69.9%) and the implications (66.2%) of precision medicine testing.

### Patients’ expectations about precision medicine

Most patients were eager to take precision medicine tests that could predict disease prognosis (92.4%), treatment response (98.1%) or drug toxicity (93.4%) (Table 5). According to patients, the principal barriers to a wider use of precision medicine test were fear of a negative impact of test results on insurability, high cost of tests and concern that their physician may give more credit to test results than their own opinion (Table 6).

**Table 5:** Interest for precision medicine testing according to patients (n = 211)

Potential benefits of precision medicine	Strongly agree or agree N (%)*	Neutral N (%)*	Disagree or strongly disagree N (%)*
Would like to take a test if it could predict the severity of my arthritis	195 (92.4)	12 (5.7)	4 (1.8)
Would like to take a test if it could predict drug efficacy	207 (98.1)	2 (0.9)	2 (1.0)
Would like to take a test if it could predict drug toxicity	197 (93.4)	9 (4.3)	5 (2.3)

%* Percentage after excluding missing values

**Table 6:** Barriers to the implementation of precision medicine according to patients

Potential barriers to implementation of precision medicine	Strongly agree or agree N (%)*	Neutral N (%)*	Disagree or strongly disagree N (%)*
Negative impact of test result on patient (n = 207)	69 (33.3)	69 (33.3)	69 (33.3)
Negative impact of test results on family members (n = 208)	74 (35.6)	66 (31.7)	68 (32.7)
Lack of confidentiality of test results (n = 211)	99 (46.9)	47 (22.3)	65 (30.8)
Negative impact of test results on job (n = 209)	63 (30.2)	60 (28.7)	86 (41.2)
Negative impact of test results on insurability (n = 209)	130 (62.2)	38 (18.2)	41 (19.6)
Discovering by accident a high risk of another disease (n = 208)	79 (38)	52 (25)	77 (37.1)
Deprivation of some treatment option due to test results (n = 209)	86 (41.1)	61 (29.2)	62 (29.7)
Doctor giving more credit to test results than patient opinion (n = 209)	102 (48.8)	65 (31.1)	42 (20.1)
Lack of reliability of test (n = 210)	99 (47)	70 (33.3)	41 (19.6)
High cost of test (n = 209)	120 (57.5)	61 (29.2)	28 (13.4)

%* Percentage after excluding missing values

### Rheumatologists/fellows and patients’ comparison

Patients expressed their greatest interest in receiving additional training on precision medicine when compared to the greatest interest of rheumatologists and fellows (97.1% and 78.1% respectively, *p* = 0.004). When comparing teaching modalities, patients were more interested in videos/podcasts (85.9% vs. 56.5%, *p* = 0.0017) and web sites (92.3% vs. 70.8%, *p* = 0.0043) whereas rheumatologists and fellows preferred conferences (96.3% vs. 51.2%, *p* < 0.0001) and small group workshops with clinical scenarios (88.5% vs. 59.1%, *p* = 0.0039). When comparing the perception of obstacles to the implementation of precision medicine in clinical practice, patients were more concerned than doctors with ensuring the confidentiality of the results (46.9% vs. 14.7%, *p* = 0.0006) and with the anxiety linked to the test results (66.7% v 52.9%, *p* = 0.1262).

## Discussion

Our study showed that Canadians living with rheumatic diseases, rheumatologists and fellows are all interested in receiving additional training in precision medicine. While convinced of the potential benefits of precision medicine tests, most physicians did not confident in their abilities in this area and perceived their training insufficient to integrate them into their clinical practice, suggesting that some physicians are confusion precision medicine tests to genetic tests only. Indeed only 42% of rheumatologists reported ordering a precision medicine test reflecting, a lack of understanding that many tests used in rheumatology are considered precision medicine tests. Although patients had less experience with precision medicine tests than physicians, they expressed greatest enthusiasm for precision medicine tests and were most confident in their ability to understand the usefulness and implications of the tests when compared to physicians. We noticed that patients a low awareness of what a precision medicine test is. Indeed, only 14.1% answered that they had ever taken such a test which is surprisingly low considering that we use those tests every day in clinical practice, suggesting a possible confusion between precision medicine tests with genetic tests. For both rheumatologists and patients, the costs of the test and their impact on future potential insurability were the major barriers to implementing precision medicine tests. Physicians were also concerned about availability and delay in obtaining test results, while patients were more concerned about the confidentiality of the results and feared that their doctor would give more credit to the test results than to their own opinion in the choice of treatment.

Comparing our results to the literature was limited due to the lack of publications in this area. There is no published study to date assessing the knowledge, experience, expectations and educational needs of rheumatologists and fellows in precision medicine. However, several studies have been conducted with primary care physicians and patients [12, 13] including a review article [8]. One Canadian study assessed perception and experience of oncologists, cardiologist and primary care physicians about personalized medicine [14]. Those studies showed results consistent with our own, demonstrating that providers generally express a positive attitude toward precision medicine testing but have modest experience and a low level of confidence in their ability to interpret and use them in a clinical setting as they feel insufficiently trained and informed about this topic. In the study published by Bonter et al. only 11% of the respondents had received formal undergraduate or postgraduate training in precision medicine and 75% would like more continuing education in this area which is congruent with our findings [14]. For patients, studies consistently demonstrate that they are highly optimistic about precision medicine and its potential for improving health, but are concerned about the risk of loss of insurability, the high costs of testing, privacy issues, and psychological harm.

Our study has several limitations. After two reminders, the number of respondents to the surveys remained lower than expected, especially for the group of rheumatologists and fellows, with the consequence that we did not meet our target sample size in order to satisfy our initial power calculation with the implication that we cannot be firm on the conclusions from our surveys. Furthermore, our study was conducted in a single country, and the results may not be generalizable to others. The patient’s survey was emailed only to members of The Arthritis Society, Arthritis Research Canada and the Arthritis Consumer Expert, so the selected patients may not be entirely representative of the entire Canadian patient population with rheumatic conditions. In addition, we did not include patients without access to electronic devices and this may have further affected the generalizability of our results. For example, we had a lower representation of patients 65 years or older when compared to the Canadian population suffering from rheumatic diseases. It is also possible that patients who are members of those associations are more interested in pursuing education in precision medicine in rheumatology than the average patient. The sample size was limited especially for rheumatology fellows with only six participants in this group. Percentage of missing data for each question varied between 10.1 and 39.4% for patients and between 2 and 39.2% for physicians, which is substantial and there were many neutral responses in the Likert scale which also affects the quality of our results. Furthermore, grouping all precision medicine tests together, including genetic tests, when evaluating the benefits and barriers to implementing precision medicine may have prevented us from detecting differences related to the different types of biomarkers available.

Our study also has strengths. It is the first study assessing knowledge, experience, expectations and educational needs of rheumatologists and rheumatology fellows in the field of precision medicine. This is a bilingual nationwide study with the participation of three patients’ associations. Our sample of participating rheumatologists, although small, was representative of Canadian rheumatologists. We evaluated the test–retest reliability of the answers to the survey, although we acknowledge that the testing was incomplete, since only some sections of the French version of the rheumatologists’ and fellows’ survey were tested while 86.3% of physicians and 74.7% of patients answering the surveys used its English version. However, there is no reason to expect the reliability to vary between the French and English version, or between French and English-speaking participants. Furthermore, our sample for reliability testing was small. Although we did not perform any qualitative study, such as focus groups, information gained was sufficient to inform the development of educational tools on precision medicine in rheumatology. Patient preference studies should be considered as a perspective of this study. Although the COVID-19 pandemic has resulted in an increase in survey-based research and improved ways of reporting survey-based studies [15], we consider that both our surveys dating from the pre-pandemic period remain applicable in the present time.

By assessing the level of knowledge and experience, expectations, principal barriers, and educational needs in precision medicine of rheumatologists, rheumatology fellows and patients with rheumatic diseases, our study will allow us to develop educational tools that address the needs reported by our participants. We will favour online continuing medical education conferences and workshops for rheumatologists and web sites or podcasts for patients. Finally, the lack of patient knowledge about precision medicine tests identified in this study reinforces the urgent need to develop shared decision-making in rheumatology clinical practice.

## Conclusions

Our study showed that patients, rheumatologists and rheumatology fellows in Canada are all interested in getting additional precision medicine education. Although most physicians are convinced of the potential benefits of precision medicine tests, the majority of those who participated in this study did not feel confident in their abilities and considered their training insufficient to integrate them into clinical practice.

## Supplementary Information

**Additional file 1.** Survey for patients (contains the English version of the survey for patients).

**Additional file 2.** Survey for rheumatologists (contains the English version of the survey for rheumatologists and fellows).

**Additional file 3.** Supplementary tables.

## Data Availability

SRG, DS and LM had full access to all the data in the study and take responsibility for the integrity of all data and accuracy of the data analysis. The datasets used and/or analysed during the current study available from the corresponding author on reasonable request.

## Abbreviations

Anti-CCP: Anti-cyclic citrullinated peptide
csDMARDs: Conventional synthetic disease-modifying anti-rheumatic drugs
CRA: Canadian Rheumatology Association
PIRA: Patients interested in research on arthritis
TPMT: Thiopurine methyltransferase
