# Supplementary material for: Expectations and educational needs of rheumatologists, rheumatology fellows and patients in the field of precision medicine in Canada, a quantitative cross-sectional and descriptive study
Source: BMC Rheumatol. 2021 Nov 29;5:52. doi: 10.1186/s41927-021-00222-2 (PMC8627786; doi:10.1186/s41927-021-00222-2)
Supplement: Supplementary file 1 — Additional file 1. Survey for patients (contains the English version of the survey for patients). [file 41927_2021_222_MOESM1_ESM.docx]

**Supplementary material**

***By answering and returning this survey, you are consenting to participate in this research project.***

**Expectations and educational needs of Canadian rheumatologists, rheumatology fellows and patients in the field of precision medicine**

A) Demographic data

1. What is your gender?

☐ Female ☐ Male ☐ I prefer not to answer

2. How old are you?

☐ 18 to 34 years old ☐ 35 to 49 years old

☐ 50 to 64 years old ☐ 65 years or older ☐ I prefer not to answer

3. What is your marital status?

☐ Single ☐ Common-law couple

☐ Married ☐ Separated/Divorced

☐ Widower ☐ I prefer not to answer

4. Do you have children?

☐ Yes ☐ No ☐ I prefer not to answer

5. What is your education level?

☐ High school (incomplete)

☐ High school diploma

☐ Diploma of College Studies (DCS/DEC)

☐ First cycle University degree (bachelor degree, first cycle doctorate)

☐ Second cycle University degree (master’s degree, DESS)

☐ Third cycle University degree (doctorate)

☐ I prefer not to answer

6. What is your field of work?

☐ Administration, business and finances

☐ Arts, culture and communication

☐ Construction, public works, landscaping and transportation

☐ Education, human and social sciences

☐ Law and public protection

☐ Natural resources, agriculture, fauna and environnement

☐ Health care

☐ Engineering

☐ Information technology

☐ Tourism and hospitality industry

☐ I am currently unemployed

☐ I am retired

☐ I prefer not to answer

7. Where do you live?

☐ Prince Edward Island

☐ Nova Scotia

☐ New Brunswick

☐ Newfoundland and Labrador

☐ Quebec

☐ Ontario

☐ Manitoba

☐ Saskatchewan

☐ Alberta

☐ British Columbia

☐ Northwest Territories

☐ Nunavut

☐ Yukon Territory

☐ I prefer not to answer

8. Which rheumatologic disease are you suffering from?

☐ Rheumatoid arthritis ☐ Psoriatic arthritis

☐ Ankylosing spondylitis ☐ Gout or pseudogout

☐ Systemic lupus erythematosus ☐ Polymyalgia rheumatica

☐ I prefer not to answer

☐ Other: Please specify __________________________________________________

9. Do you currently take medication for this disease?

☐ Yes ☐ No ☐ I prefer not to answer

10. Do you suffer from any other diseases?

☐ Yes ☐ No ☐ I prefer not to answer

11. If you answered yes to the previous question, please specify what are your other health problems. _______________________________________________________________________________

_______________________________________________________________________________

_______________________________________________________________________________

_______________________________________________________________________________

B) Introduction and definitions

Precision medicine, or personalized medicine, uses certain biological characteristics of an individual in order to offer tailored care.

In this survey, we will only use the term “precision medicine” so as to alleviate the questionnaire. This term includes all the precision medicine tests, including genetic tests, that are useful to confirm diagnosis and predict severity of the disease, drug response or side effects.

C) Experience in the field of precision medicine (including genetic tests)

12. Have you ever taken a precision medicine test?

☐ Yes ☐ No ☐ I do not know

13. If you answered yes to the previous question, please specify which precision medicine test you have taken.

_______________________________________________________________________________

_______________________________________________________________________________

_______________________________________________________________________________

14. Has anyone in your family ever taken a precision medicine test?

☐ Yes ☐ No ☐ I do not know

15. If you answered yes to the previous question, please specify which precision medicine test s/he has taken.

_______________________________________________________________________________

_______________________________________________________________________________

_______________________________________________________________________________

16. Are you confident in your ability to understand the usefulness of precision medicine tests?

☐ Yes ☐ No ☐ I do not know

17. Are you confident in your ability to understand the implications of precision medicine testing?

☐ Yes ☐ No ☐ I do not know

D) Expectations about precision medicine

18. Please indicate your degree of agreement with each of the following statements.

a) If a precision medicine test made it possible to predict the severity of my arthritis, I would like to take this test.

| Strongly agree | Agree | Neither agree nor disagree | Disagree | Strongly disagree |
| --- | --- | --- | --- | --- |

b) If a precision medicine test made it possible to predict which drug will be the most effective for treating my arthritis, I would like to take this test (for example, predictive test for response to methotrexate).

| Strongly agree | Agree | Neither agree nor disagree | Disagree | Strongly disagree |
| --- | --- | --- | --- | --- |

c) If a precision medicine test made it possible to predict which drug will give me side effects, I would like to take this test (for example, a test to predict people who will have side effects with methotrexate).

| Strongly agree | Agree | Neither agree nor disagree | Disagree | Strongly disagree |
| --- | --- | --- | --- | --- |

d) I am concerned about the impact precision medicine test results could have on me.

| Strongly agree | Agree | Neither agree nor disagree | Disagree | Strongly disagree |
| --- | --- | --- | --- | --- |

e) I am concerned about the impact precision medicine test results could have on my children and other family members.

| Strongly agree | Agree | Neither agree nor disagree | Disagree | Strongly disagree |
| --- | --- | --- | --- | --- |

f) I am concerned that the precision medicine test results could be accessible to people other than my physician.

| Strongly agree | Agree | Neither agree nor disagree | Disagree | Strongly disagree |
| --- | --- | --- | --- | --- |

g) I am concerned about the impact of unfavorable precision medicine test results on my job or my job search.

| Strongly agree | Agree | Neither agree nor disagree | Disagree | Strongly disagree |
| --- | --- | --- | --- | --- |

h) I am concerned about the impact of unfavorable precision medicine test results on my insurability.

| Strongly agree | Agree | Neither agree nor disagree | Disagree | Strongly disagree |
| --- | --- | --- | --- | --- |

i) I am concerned about the possibility of discovering by accident that I am at high risk of developing a disease like Alzheimer or cancer through precision medicine tests.

| Strongly agree | Agree | Neither agree nor disagree | Disagree | Strongly disagree |
| --- | --- | --- | --- | --- |

j) I am concerned about the possibility that precision medicine tests results will deprive me of some treatment options.

| Strongly agree | Agree | Neither agree nor disagree | Disagree | Strongly disagree |
| --- | --- | --- | --- | --- |

k) I am concerned that my doctor will give more credit to precision medicine test results than my opinion when the time comes to choose a treatment.

| Strongly agree | Agree | Neither agree nor disagree | Disagree | Strongly disagree |
| --- | --- | --- | --- | --- |

l) I am concerned about the reliability of precision medicine tests.

| Strongly agree | Agree | Neither agree nor disagree | Disagree | Strongly disagree |
| --- | --- | --- | --- | --- |

m) I am concerned about the costs of precision medicine tests.

| Strongly agree | Agree | Neither agree nor disagree | Disagree | Strongly disagree |
| --- | --- | --- | --- | --- |

E) Educational needs in the field of precision medicine

19. Would you like to receive additional information on precision medicine in rheumatology?

☐ Yes ☐ No

20. How would you like to receive additional information?

| Conferences | Yes ☐ | No ☐ |
| --- | --- | --- |
| Small group workshop with clinical scenarios | Yes ☐ | No ☐ |
| Seminar | Yes ☐ | No ☐ |
| Videos / Podcast | Yes ☐ | No ☐ |
| Self learning modules | Yes ☐ | No ☐ |
| Massive open online course (MOOC) | Yes ☐ | No ☐ |
| Web site | Yes ☐ | No ☐ |
| Other formats | Yes ☐ | No ☐ |

21. I you answered other formats to the previous question, please specify.

22. Do you have suggestions on how patient education in the field of precision medicine could be improved?

_______________________________________________________________________________

_______________________________________________________________________________

_______________________________________________________________________________

_______________________________________________________________________________

Thank you for taking the time to complete this survey. Your participation is greatly appreciated.
