# Supplementary material for: Expectations and educational needs of rheumatologists, rheumatology fellows and patients in the field of precision medicine in Canada, a quantitative cross-sectional and descriptive study
Source: BMC Rheumatol. 2021 Nov 29;5:52. doi: 10.1186/s41927-021-00222-2 (PMC8627786; doi:10.1186/s41927-021-00222-2)
Supplement: Supplementary file 2 — Additional file 2. Survey for rheumatologists (contains the English version of the survey for rheumatologists and fellows). [file 41927_2021_222_MOESM2_ESM.docx]

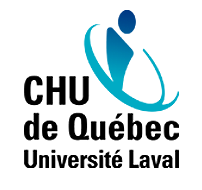


**Supplementary material**

***By answering and returning this survey, you are consenting to participate in this research project.***

**Expectations and educational needs of Canadian rheumatologists, rheumatology fellows and patients in the field of precision medicine**

A) Demographic data

1. What is your gender?

☐ Female ☐ Male ☐ I prefer not to answer

2. How old are you?

☐ 18 to 34 years old ☐ 35 to 49 years old

☐ 50 to 64 years old ☐ 65 years or older ☐ I prefer not to answer

3. If you are a rheumatology fellow, what postgraduate year are you currently enrolled in?

☐ Fourth year ☐ Fifth year

☐ Fellowship program ☐ I prefer not to answer

4. If you are a rheumatologist, how long have you been practising?

☐ Less than five years ☐ 5 to 10 years

☐ 11 to 20 years ☐ More than 20 years ☐ I prefer not to answer

5. Where do you work?

☐ Private practice

☐ Hospital

☐ University Hospital

☐ Others : Please specify

☐ I prefer not to answer

6. What is your practice environment?

☐ City ☐ Rural area ☐ I prefer not to answer

7. In wich province or territory do you work?

☐ Prince Edward Island

☐ Nova Scotia

☐ New Brunswick

☐ Newfoundland and Labrador

☐ Quebec

☐ Ontario

☐ Manitoba

☐ Saskatchewan

☐ Alberta

☐ British Columbia

☐ Northwest Territories

☐ Nunavut

☐ Yukon Territory

I prefer not to answer

8. What is your principal activity?

☐ Patient care ☐ Teaching

☐ Administration ☐ Research ☐ I prefer not to answer

B) Introduction and definitions

Precision medicine is an approach that takes into account the measurable biological characteristics of an individual in order to offer personalized care. It is sometimes called “personalized medicine”.

There are a variety of biological tests in precision medicine: diagnostic tests, prognosis tests and pharmacogenomics tests that analyze the genes of an individual so as to predict drug response and/or side effects, in order to choose the best treatment option for that person.

In this survey, we will only use the term “precision medicine” so as to alleviate the questionnaire. This term includes all the tests discussed above.

C) Experience in the field of precision medicine

9. Have you ever heard about precision medicine tests?

☐ Yes ☐ No

10. Have you ever prescribed precision medicine tests to your patients?

☐ Yes ☐ No

11. If you answered yes to the previous question, please specify which precision medicine tests you prescribed in the last year: _______________________________________________________________________________

_______________________________________________________________________________

_______________________________________________________________________________

12. Did you receive training in precision medicine as part of your university medical education?

☐ Yes ☐ No

13. Did you receive any training in precision medicine outside your university medical education?

☐ Yes ☐ No

14. If you answered yes to the previous question, in what circumstances?

☐ Medical Continuing Education Conferences

☐ By reading articles in scientific journals

☐ By reading on the internet

☐ Discussion with colleagues

☐ Others

15. Do you think your precision medicine training is sufficient to integrate the tests in your current medical practice?

☐ Yes ☐ No

D) Clinical Vignettes

Please read carefully the clinical vignettes below and answer the questions.

Case 1

A 37-year-old man has inflammatory back pain with morning stiffness lasting 90 minutes every day and nocturnal awakenings. Pain is not relieved by the Naproxen prescribed by his family doctor. There are no peripheral arthralgia, enthesitis, uveitis, dactylitis, psoriasis or gastrointestinal symptoms. His mother has Crohn’s disease. The physical examination is normal except for a slight limitation of the mobility of the lumbar spine.

16. Would you immediately prescribe a HLA-B27 test for this patient?

☐ Yes ☐ No ☐ I do not know

17. In your work place, what kind of HLA-B27 test is done?

☐ Phenotypic test

☐ Genotyping

☐ Phenotypic test first and then genotyping if test result is uncertain

☐ I do not know

You tell your patient that he is positive for HLA-B27. He is really worried about his three kids inheriting the HLA-B27 gene and developing ankylosing spondylitis. He asks you to test his children ages 13, 10 and 7 for the HLA-B27 gene.

18. Would you prescribe the HLA-B27 test to his children?

☐ Yes ☐ No ☐ I do not know

19. Please explain your answer: ________________________________________________________

_______________________________________________________________________________

_______________________________________________________________________________

_______________________________________________________________________________

_______________________________________________________________________________

Case 2

A 34-year-old woman has experienced symmetrical inflammatory polyarthralgia of PIP, MCP, wrists and MTP for the past five months without any other symptoms. The physical examination reveals synovitis of the PIPs, MCPs, wrists and MTPs. You order basic blood tests, X-rays and dosage of the inflammatory markers, rheumatoid factor and anti-cyclic citrullinated peptide (anti-CCP) antibody.

20. Why would you use the anti-CCP antibodies test for this patient? Select all the answers that apply.

☐ Confirm the rheumatoid arthritis diagnosis

☐ Determine the prognosis

☐ Predict the efficacy of some biologic drugs and help choose the treatment

☐ Predict the toxicity risk of some drugs

☐ I do not know

Case 3

A 34-year-old woman with a myositis-related antisynthetase syndrome comes in for a follow up visit. Despite taking subcutaneous methotrexate at maximum dose, there is a recurrence of weaknesses and an increase in CK while tapering off prednisone. You increase her prednisone and change her treatment from methotrexate to azathioprine.

21. Would you order a thiopurine methyltransferase (TPMT) test before starting treatment with azathioprine?

☐ Yes ☐ No ☐ I do not know

22. In your work place, what type of TPMT test is done?

☐ Enzyme activity test

☐ Genotyping

☐ I do not know

23. Why would you order a TPMT test? Select all the answers that apply.

☐ Identify patients who are likely to respond to azathioprine

☐ Identify patients who are unlikely to respond to azathioprine

☐ Identify patients who are likely to develop neutropenia associated with azathioprine use

☐ I do not know

Case 4

A 76-year-old Thai man has recurring episodes of gout. His medical history is positive for coronary heart disease with coronary bypass 10 years ago, type 2 diabetes, and stage G3a chronic kidney disease with a creatinine clearance of 50ml/min. His acute gout attacks are successfully treated with colchicine, which was prescribed by his primary care physician. He has three to four acute attacks each year. On physical examination there are no tophi.

You order blood tests and receive the results below.

- CBC: normal
- Creatinine: 130 with creatinine clearance of 49 ml/min
- Uric acid: 536

You want to prescribe allopurinol to this patient.

24. Would you order a HLA–B* 5801 test for this patient?

☐ Yes ☐ No ☐ I do not know

25. For which of the following populations do the 2012 American College of Rheumatology guidelines on gout management advise to perform HLA–B* 5801 genetic test before starting allopurinol? Select all the answers that apply.

☐ All the patients ☐ Patients with stage G3a chronic kidney disease or worse

☐ Thai ☐ African

☐ Han Chinese ☐ Patients 75 years or older

☐ Korean with stage G3a chronic kidney disease or worse

☐ I do not know

26. Why would you order a HLA-B* 5801 genetic test? Select all the answers that apply.

☐ Identify patients who will need higher dosage of allopurinol to reach efficacy

☐ Identify patients who are unlikely to respond to allopurinol

☐ Identify patients who are likely to develop a hypersensitivity reaction to allopurinol

☐ I do not know

Case 5

You want to prescribe a newly available biologic drug to a patient with rheumatoid arthritis. This new drug comes with a companion diagnostic that can identify patients in whom the therapy is likely to provide maximum benefit in less than 3 months, but only in 55% of patients.

You decide to do the diagnostic test, which results could be reported in different ways.

27. Which reporting method are you most comfortable with? Please classify the items in the order of your preferences.

☐ Fagan nomogram (graph that compares pre-test and post-test probabilities)

☐ Dichotomous result, for example: “positive/negative result”

☐ Risk categories: for example, low risk of response to a specific drug, moderate/uncertain risk of response, high risk of response.

☐ Other reporting method

28. If you answered other reporting method to the previous question, please specify.

E) Knowledge in the field of precision medicine

29. Please indicate your degree of agreement with each of the following statements about your knowledge of precision medicine.

a) I am able to identify the clinical situations in which precision medicine tests are useful.

| Strongly agree | Agree | Neither agree nor disagree | Disagree | Strongly disagree |
| --- | --- | --- | --- | --- |

b) I am well aware of the precision medicine tests available in my work place.

| Strongly agree | Agree | Neither agree nor disagree | Disagree | Strongly disagree |
| --- | --- | --- | --- | --- |

c) I am comfortable with prescribing precision medicine tests.

| Strongly agree | Agree | Neither agree nor disagree | Disagree | Strongly disagree |
| --- | --- | --- | --- | --- |

d) I have confidence in my ability to provide quality counseling to my patients about the risks, benefits and limitations of precision medicine testing.

| Strongly agree | Agree | Neither agree nor disagree | Disagree | Strongly disagree |
| --- | --- | --- | --- | --- |

e) I am comfortable with interpreting precision medicine test results.

| Strongly agree | Agree | Neither agree nor disagree | Disagree | Strongly disagree |
| --- | --- | --- | --- | --- |

f) I have confidence in my ability to communicate the results of the precision medicine tests to my patients and to answer their questions.

| Strongly agree | Agree | Neither agree nor disagree | Disagree | Strongly disagree |
| --- | --- | --- | --- | --- |

g) I am comfortable with recommending a treatment based on precision medicine tests results.

| Strongly agree | Agree | Neither agree nor disagree | Disagree | Strongly disagree |
| --- | --- | --- | --- | --- |

F) Expectations about precision medicine

39. Please indicate your degree of agreement with each of the following statements about the potential benefits of precision medicine tests.

a) I think that precision medicine tests are relevant to my current medical practice.

| Strongly agree | Agree | Neither agree nor disagree | Disagree | Strongly disagree |
| --- | --- | --- | --- | --- |

b) I think that precision medicine tests results are susceptible to change my approach.

| Strongly agree | Agree | Neither agree nor disagree | Disagree | Strongly disagree |
| --- | --- | --- | --- | --- |

c) I think that precision medicine tests are useful to determine a patient’s prognosis.

| Strongly agree | Agree | Neither agree nor disagree | Disagree | Strongly disagree |
| --- | --- | --- | --- | --- |

d) I think that precision medicine tests can be useful diagnostic tools.

| Strongly agree | Agree | Neither agree nor disagree | Disagree | Strongly disagree |
| --- | --- | --- | --- | --- |

e) I think that precision medicine tests are likely to help with choosing the most effective first-line treatments (e.g.: predictive test of methotrexate efficacy)

| Strongly agree | Agree | Neither agree nor disagree | Disagree | Strongly disagree |
| --- | --- | --- | --- | --- |

f) I think that precision medicine tests can help minimise the side effects (toxicity) of the treatments (e.g.: predictive test of methotrexate toxicity)

| Strongly agree | Agree | Neither agree nor disagree | Disagree | Strongly disagree |
| --- | --- | --- | --- | --- |

g) I think that precision medicine tests can improve patients’ compliance to treatment.

| Strongly agree | Agree | Neither agree nor disagree | Disagree | Strongly disagree |
| --- | --- | --- | --- | --- |

31. Please indicate your degree of agreement with each of the following statements about the potential barriers to implementation of precision medicine tests in medical practice.

a) I think that there is not enough scientific evidence to support the use of precision medicine tests in current medical practice.

| Strongly agree | Agree | Neither agree nor disagree | Disagree | Strongly disagree |
| --- | --- | --- | --- | --- |

b) I think that the lack of guidelines on precision medicine is a barrier to its clinical implementation.

| Strongly agree | Agree | Neither agree nor disagree | Disagree | Strongly disagree |
| --- | --- | --- | --- | --- |

c) I think that limited accessibility to precision medicine tests is a barrier to their implementation in medical practice.

| Strongly agree | Agree | Neither agree nor disagree | Disagree | Strongly disagree |
| --- | --- | --- | --- | --- |

d) I think that the cost of precision medicine tests is a barrier to their implementation in medical practice.

| Strongly agree | Agree | Neither agree nor disagree | Disagree | Strongly disagree |
| --- | --- | --- | --- | --- |

e) I think that the delay to obtain precision medicine test results is a barrier to their implementation in medical practice.

| Strongly agree | Agree | Neither agree nor disagree | Disagree | Strongly disagree |
| --- | --- | --- | --- | --- |

f) I think that my knowledge is insufficient to use precision medicine tests in my current medical practice.

| Strongly agree | Agree | Neither agree nor disagree | Disagree | Strongly disagree |
| --- | --- | --- | --- | --- |

g) I am concerned about the confidentiality of the precision medicine test results.

| Strongly agree | Agree | Neither agree nor disagree | Disagree | Strongly disagree |
| --- | --- | --- | --- | --- |

h) I am concerned about the impact of precision medicine test results on the patients’ job or job search.

| Strongly agree | Agree | Neither agree nor disagree | Disagree | Strongly disagree |
| --- | --- | --- | --- | --- |

i) I am concerned about the impact of precision medicine test results on the patients’ insurability.

| Strongly agree | Agree | Neither agree nor disagree | Disagree | Strongly disagree |
| --- | --- | --- | --- | --- |

j) I am concerned about the anxiety patients might feel because of the precision medicine test results.

| Strongly agree | Agree | Neither agree nor disagree | Disagree | Strongly disagree |
| --- | --- | --- | --- | --- |

k) I am concerned about the impact of precision medicine test results on my patients’ family members.

| Strongly agree | Agree | Neither agree nor disagree | Disagree | Strongly disagree |
| --- | --- | --- | --- | --- |

G) Educational needs in the field of precision medicine

32. Would you like to receive additional training on precision medicine in rheumatology?

☐ Yes ☐ No

33. If you answered no to the previous question, please specify why you would not want additional training? ____________________________________________________________________________________________________________________________________________________________________________________________________________________________________________________________________________________________________________________________

34. If you answered yes, would you like additional training on the following topics?

| Tests indications | Yes ☐ | No ☐ |
| --- | --- | --- |
| Clinical utility | Yes ☐ | No ☐ |
| How to prescribe the tests | Yes ☐ | No ☐ |
| Test validity and accuracy | Yes ☐ | No ☐ |
| How to interpret the test results | Yes ☐ | No ☐ |
| Legal, ethical and social implications | Yes ☐ | No ☐ |
| Cost-Benefit assessment | Yes ☐ | No ☐ |
| Strategies for integrating the tests into healthcare practice | Yes ☐ | No ☐ |
| Other subjects. | Yes ☐ | No ☐ |

35. I you answered other subjects, please specify: __________________________________________________

36. How would you like to receive additional training?

| Conferences | Yes ☐ | No ☐ |
| --- | --- | --- |
| Small group workshop with clinical scenarios | Yes ☐ | No ☐ |
| Seminar | Yes ☐ | No ☐ |
| Videos / Podcast | Yes ☐ | No ☐ |
| Self learning modules | Yes ☐ | No ☐ |
| Massive open online course (MOOC) | Yes ☐ | No ☐ |
| Web site | Yes ☐ | No ☐ |
| Other formats | Yes ☐ | No ☐ |

37. If you answered other formats, please specify: __________________________________________________

38. Would you prefer a combination of training methods?

☐ Yes ☐ No

39. Do you have suggestions on how training in the field of precision medicine could be improved?

_______________________________________________________________________________

_______________________________________________________________________________

_______________________________________________________________________________

_______________________________________________________________________________

Thank you for taking the time to complete this survey. Your participation is greatly appreciated.
