# Supplementary material for: Expectations and educational needs of rheumatologists, rheumatology fellows and patients in the field of precision medicine in Canada, a quantitative cross-sectional and descriptive study
Source: BMC Rheumatol. 2021 Nov 29;5:52. doi: 10.1186/s41927-021-00222-2 (PMC8627786; doi:10.1186/s41927-021-00222-2)
Supplement: Supplementary file 3 — Additional file 3. Supplementary tables. [file 41927_2021_222_MOESM3_ESM.doc]

**Supplementary material**

**Supplementary Table 1** - Demographic characteristics of participating rheumatologists and fellows (N = 51)

| **Characteristics** | **N (%)*** |
| --- | --- |
| Sex   - Female - Male - I prefer not to answer | 22/49 (44.9)  25/49 (51.0)  2/49 (4.1) |
| Age   - 18 to 34 years old - 35 to 49 years old - 50 to 64 years old - 65 years or older - I prefer not to answer | 7/50 (14.0)  20/50 (40.0)  11/50 (22.0)  10/50 (20.0)  2/50 (4.0) |
| Postgraduate year (fellows only)   - Fourth year - Fifth year - Fellowship program - I prefer not to answer | 1/6 (16.7)  2/6 (33.3)  1/6 (16.7)  2/6 (33.3) |
| Practice year (rheumatologists only)   - Less than five years - 5 to 10 years - 11 to 20 years - More than 20 years - I prefer not to answer | 8/45 (17.8)  7/45 (15.6)  12/45 (26.7)  16/45 (35.6)  2/45 (4.4) |
| Workplace †   - Private practice - Hospital - University hospital - Other | 20/50 (40.0)  6/50 (12.0)  32/50 (64.0)  1/50 (2.0) |
| Practice environment   - City - Rural | 47/50 (94.0)  3/50 (6.0) |
| Province   - New Brunswick - Newfoundland and Labrador - Quebec - Ontario - Alberta - British Columbia - I prefer not to answer | 1/50 (2.0)  1/50 (2.0)  11/50 (22.0)  8/50 (16.0)  9/50 (18.0)  18/50 (36.0)  2/50 (4.0) |
| Principal activity   - Patient care - Administration - Research - I prefer not to answer | 43/50 (86.0)  1/50 (2.0)  4/50 (8.0)  2/50 (4.0) |

%* Percentage after excluding missing values

† not mutually exclusive

**Supplementary Table 2** - Representativeness of the Rheumatologists and fellows’ sample

| **Demographic characteristic** | **Proportion in our sample,%*** | **Proportion in the reference population,%*** | **P-value** |
| --- | --- | --- | --- |
| Female | 46.8 (95% CI : 32.1-61.9) | 53 | 0.3951 |
| Age <50 years old | 56.3 (95% CI:41.2-70.5) | 50 | 0.3865 |
| Practice years - less than 5 | 18.6 (95% CI:8.4-33.4) | 25 | 0.3328 |
| Practice years - 5 to 10 years | 16.3 (95% CI:6.8-30.7) | 12 | 0.3879 |
| Practice years -11 to 20 years | 27.9 (95% CI:15.4-43.7) | 23 | 0.4445 |
| Practice years - more than 20 | 37.2 (95% CI : 23.0-53.3) | 40 | 0.7087 |
| British Columbia | 37.5 (95% CI : 24.0-52.7) | 15 | <0.0001 |
| Alberta | 18.8 (95% CI : 9.0-32.6) | 20 | 0.8286 |
| Ontario | 16.7 (95% CI:7.5-30.2) | 38 | 0.0023 |
| Quebec | 22.9 (95% CI : 12.0-37.3) | 19 | 0.4891 |
| Atlantic provinces | 4.2 (95% CI : 0.5-14.3) | 7 | 0.4417 |

%* Percentage after excluding missing values

**Supplementary table 3** – Reliability of results for rheumatologists and fellows’ survey

| **Question** | **Kappa coefficient** | **95% Confidence Interval** |
| --- | --- | --- |
| Question 1 - Knowledge 1 | 1.0 | 1.0 - 1.0 |
| Question 2 - Knowledge 2 | 0.0 | 0.0 - 0.0 |
| Question 3 - Knowledge 3 | 0.5714 | 0.3755 - 0.7674 |
| Question 4 - Knowledge 4 | 0.0 | 0.0 - 0.0 |
| Question 5 - Knowledge 5 | -0.5 | -1.0 - 0.1001 |
| Question 6 - Knowledge 6 | 0.4 | -0.3681 - 1.0 |
| Question 7 - Knowledge 7 | 0.5714 | 0.0530 - 1.0 |
| Question 8 - Expectations 1 | 0.2500 | -0.1469 - 0.6469 |
| Question 9 - Expectations 2 | 1.0 | 1.0 - 1.0 |
| Question 10 - Expectations 3 | 1.0 | 1.0 |
| Question 11 - Expectations 4 | 1.0 | 1.0 |
| Question 12 - Expectations 5 | 1.0 | 1.0 - 1.0 |
| Question 13 - Expectations 6 | 1.0 | 1.0 - 1.0 |
| Question 14 - Expectations 7 | 0.4 | -0.3681 - 1.0 |
| Question 15 - Expectations 8 | 0.5714 | 0.0530 - 1.0 |
| Question 16 - Expectations 9 | 1.0 | 1.0 - 1.0 |
| Question 17 - Expectations 10 | 0.0 | 0.0 - 0.0 |
| Question 18 - Expectations 11 | 0.0 | 0.0 - 0.0 |
| Question 19 - Expectations 12 | 0.0 | 0.0 - 0.0 |
| Question 20 - Expectations 13 | -0.2857 | -0.6251 - 0.0537 |
| Question 21 - Expectations 14 | 0.5714 | 0.0530 - 1.0 |
| Question 22 - Expectations 15 | -0.2857 | -0.6251 - 0.0537 |
| Question 23 - Expectations 16 | 0.4 | -0.3681 - 1.0 |
| Question 24 - Expectations 17 | 0.5714 | 0.0530 - 1.0 |
| Question 25 - Expectations 18 | 0.4 | -0.3681 – 1.0 |

**Supplementary table 4** - Topics of interest for additional training in precision medicine according to rheumatologists and fellows

| **Topics** | **N (%)*** |
| --- | --- |
| Clinical utility | 25/25 (100) |
| Test validity and accuracy | 24/24 (100) |
| Tests indications | 24/25 (96.0) |
| How to interpret the test results | 24/24 (100) |
| Strategies for integrating the tests into health care practice | 23/23 (100) |
| Cost-benefit assessment | 23/24 (95.8) |
| How to prescribe the tests | 22/23 (95.7) |
| Legal, ethical and social implications | 22/23 (95.7) |
| Other subjects | 1/7 (14.3) |

%* Percentage after excluding missing values

**Supplementary table 5** - Teaching methods for additional training in precision medicine according to rheumatologists and fellows

| **Topics** | **N (%)*** |
| --- | --- |
| Conferences | 26/27 (96.3) |
| Small group workshop with clinical scenarios | 23/26 (88.5) |
| Self-learning modules | 19/24 (79.2) |
| Web site | 17/24 (70.8) |
| Seminar | 14/22 (63.6) |
| Videos/Podcast | 23/41 (56.5) |
| Massive open online course (MOOC) | 10/22 (45.5) |
| Other formats | 1/12 (8.3) |

%* Percentage after excluding missing values

**Supplementary Table 6** - Demographic characteristics of participating patients (N = 277)

| **Characteristics** | **N (%)*** |
| --- | --- |
| Sex   - Female - Male | 199/238 (83.6)  39/238 (16.4) |
| Age   - 18 to 34 years old - 35 to 49 years old - 50 to 64 years old - 65 years or older - I prefer not to answer | 11/239 (4.6)  48/239 (20.1)  108/239 (45.2)  71/239 (29.7)  1/239 (0.4) |
| Marital status   - Single - Common-law couple - Married - Separated/Divorced - Widower - I prefer not to answer | 29/238 (12.2)  21/238 (8.8)  138/238 (58.0)  37/238 (15.5)  11/238 (4.6)  2/238 (0.8) |
| Children   - Yes - No - I prefer not to answer | 176/238 (73.9)  59/238 (24.8)  3/238 (1.3) |
| Education level   - High school (incomplete) - High school diploma - Diploma of College Studies (DCS/DEC) - University degree - I prefer not to answer | 5/237 (2.1)  30/237 (12.7)  71/237 (30)  129/237 (54.4)  2/237 (0.8) |
| Field of work   - Administration, business and finances - Arts, culture and communication - Construction, public works, landscaping and transportation - Education, human and social sciences - Law and public protection - Natural resources, agriculture, fauna and environment - Health care - Engineering - Information technology - Tourism and hospitality industry - I am currently unemployed - I am retired - I prefer not to answer | 30/239 (12.6)  6/239 (2.5)  3/239 (1.3)  21/239 (8.8)  5/239 (2.1)  2/239 (0.8)  47/239 (19.7)  1/239 (0.4)  7/239 (2.9)  2/239 (0.8)  16/239 (6.7)  94/239 (39.3)  5/239 (2.1) |
| Province   - Prince Edward Island - Nova Scotia - New Brunswick - Newfoundland and Labrador - Quebec - Ontario - Manitoba - Saskatchewan - Alberta - British Columbia - I prefer not to answer | 1/239 (0.4)  5/239 (2.1)  7/239 (2.9)  1/239 (0.4)  80/239 (33.5)  64/239 (26.8)  10/239 (4.2)  5/239 (2.1)  18/239 (7.5)  46/239 (19.2)  2/239 (0.8) |
| Rheumatic disease   - Rheumatoid arthritis - Psoriatic arthritis - Ankylosing spondylitis - Gout or pseudogout - Systemic lupus erythematosus - Polymyalgia rheumatica - I prefer not to answer - Other | 131/272 (48.2)  35/272 (12.9)  56/272 (20.6)  6/272 (2.2)  8/272 (2.9)  3/272 (1.3)  2/272 (0.7)  31/272 (11.4) |
| Medication for rheumatic disease   - Yes - No | 207/234 (88.5)  27/234 (11.5) |
| Comorbidities   - Yes - No - I prefer not to answer | 139/229 (60.7)  87/229 (38.0)  3/229 (1.3) |

%* Percentage after excluding missing values

**Supplementary Table 7** - Representativeness of the patients’ sample

| **Demographic characteristic** | **Proportion in our sample,%*** | **Proportion in the reference population,%*** | **P-value** |
| --- | --- | --- | --- |
| Female | 83.6 (95% CI : 78.3-88.1) | 64 | <0.0001 |
| Age ≥ 65 years old | 25.7 (95% CI : 20.7-31.3) | 42 | <0.0001 |
| British Columbia | 19.4 (95% CI : 14.6-25.0) | 13.2 | 0.0045 |
| Alberta | 7.6 (95% CI : 4.6-11.7) | 9.7 | 0.2798 |
| Saskatchewan | 2.1 (95% CI:0.7-4.9) | 3.3 | 0.3050 |
| Manitoba | 4.2 (95% CI:2.0-7.6) | 3.7 | 0.6528 |
| Ontario | 27.0 (95% CI:21.5-33.1) | 42.8 | <0.0001 |
| Quebec | 33.8 (95% CI:27.8-40.2) | 17.5 | <0.0001 |
| Newfoundland and Labrador | 0.4 (95% CI : 0.0-2.3) | 2.1 | 0.0726 |
| New Brunswick | 3.0 (95% CI : 1.2-6.0) | 2.9 | 0.9828 |
| Nova Scotia | 2.1 (95% CI : 0.7-4.9) | 4.2 | 0.1126 |
| Prince Edward Island | 0.4 (95% CI : 0.0-2.3) | 0.5 | 0.8491 |

%* Percentage after excluding missing values

**Supplementary table 8** - Reliability of results for patients’ survey

| **Question** | **Kappa coefficient** | **95% Confidence Interval** |
| --- | --- | --- |
| Question 1 - Sex | 1.0 | 1.0 - 1.0 |
| Question 2 - Age | 1.0 | 1.0 – 1.0 |
| Question 3 - Marital status | 1.0 | 1.0 - 1.0 |
| Question 4 - Children | 1.0 | 1.0 - 1.0 |
| Question 5 - Education | 1.0 | 1.0 - 1.0 |
| Question 6 - Work | 0.3333 | -0.3200 - 0.9867 |
| Question 7 - Province | 1.0 | 1.0 - 1.0 |
| Question 8 - Rheumatic disease | 0.3333 | -0.3200 - 0.9867 |
| Question 9 - Medication | 0.5 | -0.2350 - 1.0 |
| Question 10 - Comorbidities | 1.0 | 1.0 - 1.0 |
| Question 11 - Experience 1 | No kappa coefficient since the sample size equals 1 | |
| Question 12 - Experience 2 | 1.0 | 1.0 - 1.0 |
| Question 13 - Experience 3 | 0.0 | 0.0 - 0.0 |
| Question 14 - Experience 4 | 0.0 | 0.0 - 0.0 |
| Question 15 - Expectations 1 | 0.6 | 0.0401 - 1.0 |
| Question 16 - Expectations 2 | 0.7143 | 0.1912 - 1.0 |
| Question 17 - Expectations 3 | 0.6667 | 0.2895 - 1.0 |
| Question 18 - Expectations 4 | 0.4286 | 0.1820 - 0.6751 |
| Question 19 - Expectations 5 | 0.5714 | 0.3417 - 0.8012 |
| Question 20 - Expectations 6 | 0.5 | 0.1633 - 0.8367 |
| Question 21 - Expectations 7 | 0.6364 | 0.4503 - 0.8225 |
| Question 22 - Expectations 8 | 0.6 | 0.0401 - 1.0 |
| Question 23 - Expectations 9 | 1.0 | 1.0 - 1.0 |
| Question 24 - Expectations 10 | 1.0 | 1.0 - 1.0 |
| Question 25 - Expectations 11 | 0.3333 | -0.0439 - 0.7105 |
| Question 26 - Expectations 12 | 1.0 | 1.0 - 1.0 |
| Question 27 - Expectations 13 | 1.0 | 1.0 - 1.0 |
| Question 28 - Educational needs 1 | 1.0 | 1.0 - 1.0 |
| Question 29 - Educational needs 2 | 1.0 | 1.0 - 1.0 |
| Question 30 - Educational needs 3 | 1.0 | 1.0 - 1.0 |
| Question 31 - Educational needs 4 | 0.0 | 0.0 - 0.0 |
| Question 32 - Educational needs 5 | 1.0 | 1.0 - 1.0 |
| Question 33 - Educational needs 6 | 1.0 | 1.0 - 1.0 |
| Question 34 - Educational needs 7 | 1.0 | 1.0 - 1.0 |
| Question 35 - Educational needs 8 | 1.0 | 1.0 - 1.0 |

**Supplementary table 9** - Teaching methods for additional training in precision medicine according to patients

| **Topics** | **N (%)*** |
| --- | --- |
| Web site | 181/196 (92.3) |
| Videos/Podcast | 152/177 (85.9) |
| Self-learning modules | 146/175 (83.4) |
| Seminar | 108/169 (63.9) |
| Massive open online course (MOOC) | 104/168 (61.9) |
| Small group workshop with clinical scenarios | 101/171 (59.1) |
| Conferences | 86/168 (51.2) |
| Other formats | 39/117 (33.3) |

%* Percentage after excluding missing values
